# Differential clustering of visual and choice- and saccade-related activity in macaque V3A and CIP

**DOI:** 10.1152/jn.00285.2023

**Published:** 2024-03-13

**Authors:** Zikang Zhu, Byounghoon Kim, Raymond Doudlah, Ting-Yu Chang, Ari Rosenberg

**Affiliations:** ^1^Department of Neuroscience, School of Medicine and Public Health, University of Wisconsin–Madison, Madison, Wisconsin, United States; ^2^School of Medicine, National Defense Medical Center, Taipei, Taiwan

**Keywords:** choice signals, neuronal clustering, oculomotor, sensorimotor, vision

## Abstract

Neurons in sensory and motor cortices tend to aggregate in clusters with similar functional properties. Within the primate dorsal (“where”) pathway, an important interface between three-dimensional (3-D) visual processing and motor-related functions consists of two hierarchically organized areas: V3A and the caudal intraparietal (CIP) area. In these areas, 3-D visual information, choice-related activity, and saccade-related activity converge, often at the single-neuron level. Characterizing the clustering of functional properties in areas with mixed selectivity, such as these, may help reveal organizational principles that support sensorimotor transformations. Here we quantified the clustering of visual feature selectivity, choice-related activity, and saccade-related activity by performing correlational and parametric comparisons of the responses of well-isolated, simultaneously recorded neurons in macaque monkeys. Each functional domain showed statistically significant clustering in both areas. However, there were also domain-specific differences in the strength of clustering across the areas. Visual feature selectivity and saccade-related activity were more strongly clustered in V3A than in CIP. In contrast, choice-related activity was more strongly clustered in CIP than in V3A. These differences in clustering may reflect the areas’ roles in sensorimotor processing. Stronger clustering of visual and saccade-related activity in V3A may reflect a greater role in within-domain processing, as opposed to cross-domain synthesis. In contrast, stronger clustering of choice-related activity in CIP may reflect a greater role in synthesizing information across functional domains to bridge perception and action.

**NEW & NOTEWORTHY** The occipital and parietal cortices of macaque monkeys are bridged by hierarchically organized areas V3A and CIP. These areas support 3-D visual transformations, carry choice-related activity during 3-D perceptual tasks, and possess saccade-related activity. This study quantifies the functional clustering of neuronal response properties within V3A and CIP for each of these domains. The findings reveal domain-specific cross-area differences in clustering that may reflect the areas’ roles in sensorimotor processing.

## Graphical abstract

**Figure:**
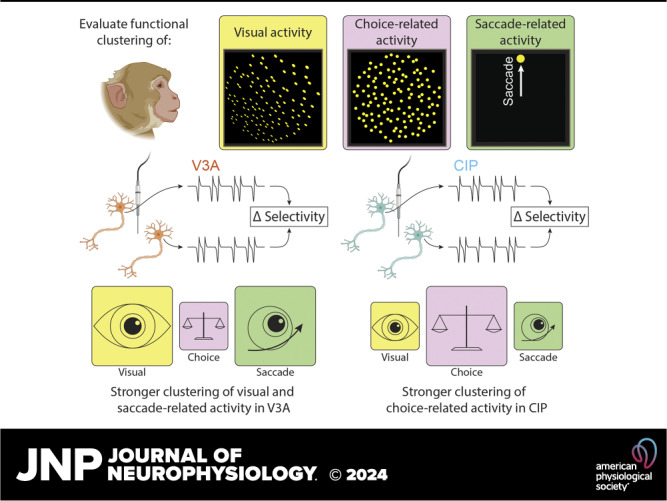

## INTRODUCTION

The anatomical clustering of neurons with similar functional properties is thought to support efficient neural computation through the optimization of axonal wiring (1–7). Such organization, referred to as functional clustering, is widely observed in sensory and motor cortex. For instance, primary visual cortex shows clustering of orientation selectivity (8), posterior inferior temporal cortex shows clustering of color selectivity (9), and primary motor cortex shows clustering of arm reach directions (10). Whether such clustering occurs within posterior parietal cortex (PPC), which supports integrative and sensorimotor functions (11, 12), has been less explored. Notably, PPC neurons are often selective for multiple sensory modalities and/or effectors (13–18), which may support high-dimensional representations that enable diverse and flexible behavioral repertoires (19–21). This mixed selectivity may conceivably influence functional clustering. Previous work revealed clustering of visual and vestibular self-motion cues as well as choice-related activity in the ventral intraparietal area, but whether mixed selectivity moderated that clustering was not tested (22, 23). Characterizing the clustering of neuronal response properties in PPC and how this relates to mixed selectivity may help reveal organizational principles that constrain the implementation of sensorimotor transformations. To this end, the present study quantified and contrasted the clustering of visual feature selectivity, choice-related activity, and saccade-related activity in area V3A and the caudal intraparietal (CIP) area of macaque monkeys.

Areas V3A and CIP are hierarchically organized neighboring brain regions that bridge visual and parietal cortex (24). Area V3A receives direct input from V1 and V2 (25, 26) and projects to CIP (27). The areas are implicated in the transformation of retinal images into three-dimensional (3-D) representations of object pose (position and orientation) and carry choice-related activity during 3-D visual discrimination tasks (28–36). Both areas contain neurons whose visual responses are modulated by extraretinal signals (37–42). Saccade-related activity predicting the direction and timing of eye movements is similarly prevalent in the two areas but begins earlier in V3A (35, 36). Neurons in both areas also form sensorimotor associations between their visual and saccade direction preferences, which are stronger in CIP. These associations may facilitate goal-directed behaviors through CIP’s downstream projections to oculomotor and prehensile areas (27, 43–46).

To assess functional clustering, we compared the response properties of pairs of well-isolated single neurons simultaneously recorded with tetrodes. Visual feature selectivity, choice-related activity, and saccade-related activity clustered in both areas. However, there were also domain-specific, cross-area differences in the strength of clustering. Visual selectivity, which was measured by presenting planar surfaces at different 3-D poses, was more strongly clustered in V3A. In contrast, choice-related activity measured during a 3-D orientation discrimination task (47) was more strongly clustered in CIP. Intriguingly, saccade-related activity measured during a visually guided saccade task (48, 49) was more strongly clustered in V3A. The strength of clustering had little to no dependence on whether the neurons showed mixed selectivity. Given that functional clustering is thought to facilitate computations within the clustered feature space, these findings are consistent with V3A having a stronger role in the within-domain processing of visual and saccade-related signals. They are further consistent with CIP having a stronger role in the cross-domain synthesis of sensory and oculomotor signals. The differential clustering of visual and choice- and saccade-related activity in V3A and CIP may reflect the areas’ functional contributions to sensorimotor processing.

## MATERIALS AND METHODS

### Animal Preparation

As previously described in detail, the experiments were performed with three male rhesus monkeys (*Macaca mulatta*) (35, 36). Briefly, the monkeys were implanted with a Delrin ring for head restraint and a removable recording grid for guiding electrodes. After recovery, they were trained to sit in a primate chair with head restraint and to fixate visual targets within 2° version and 1° vergence windows for liquid rewards. All procedures followed the National Institutes of Health *Guide for the Care and Use of Laboratory Animals* and were approved by the Institutional Animal Care and Use Committee (IACUC) at the University of Wisconsin–Madison.

### Experimental Control and Stimulus Presentation

Experimental control was performed with REC-GUI software (RRID:SCR_019008) (50). Stimuli were rendered with Psychtoolbox 3 (MATLAB R2016b; NVIDIA GeForce GTX 970) and rear projected onto a polarization preserving screen (Stewart Film Screen, Inc.) with a DLP LED projector (PROPixx; VPixx Technologies, Inc.). The pixel resolution was 1,280 × 720 (70° × 43° of visual angle), and the frame rate was 240 Hz (120 Hz/eye). The screen distance was 57 cm. A circular polarizer and polarized glasses were used for stereoscopic presentation. A phototransistor circuit was used to align neuronal responses to the stimulus onset. Eye tracking was performed optically at 1 kHz (EyeLink 1000 Plus; SR Research).

### Visual Stimuli

The visual stimuli were planar surfaces defined by 250 nonoverlapping dots uniformly distributed across the plane in world coordinates. The planes were 20° in diameter and presented at screen center. The dots were rendered with stereoscopic and perspective cues. Orientation was parameterized as slant and tilt (32, 51). All combinations of four slants (15° to 60°, 15° steps) and eight tilts (0° to 315°, 45° steps) plus the frontoparallel plane (slant = 0°, tilt undefined) were presented. Each orientation was presented at four distances (37, 57, 97, and 137 cm). The fixation target was always at screen distance (57 cm).

### Behavioral Tasks

#### Tilt discrimination task.

The monkeys performed an eight-alternative forced-choice tilt discrimination task during the presentation of the planar surfaces (47). On each trial, fixation was held on a target at the center of the screen for 300 ms. A plane then appeared for 1,000 ms while fixation remained on the target. The fixation target and plane then disappeared, and eight choice targets corresponding to the eight tilts appeared (polar angles: 0° to 315°, 45° steps; 11° eccentricity). A saccade was made to the target at the side of the plane perceived as nearest (e.g., the left target for a left-near plane). A liquid reward was given for correct responses. Responses to frontoparallel planes (task ambiguous) were pseudorandomly rewarded.

#### Visually guided saccade task.

The monkeys also performed a visually guided saccade task (48, 49). On each trial, fixation was held on a target at the center of the screen for 1,300 ms. The fixation target then disappeared, and a saccade target appeared at one of eight locations matching the choice target locations in the tilt discrimination task. A saccade to that target was made in exchange for a liquid reward.

Trials of the two tasks were interleaved. If fixation was prematurely broken or a response was not made within 500 ms of the choice/saccade targets appearing, the trial was aborted and reshuffled into the remaining trials.

### Neuronal Recordings

The areas were identified based on magnetic resonance imaging scans, gray/white matter transitions, and functional properties (32–36). A total of 91 V3A and 53 CIP recording sessions were performed with linear array probes with either four or eight tetrodes separated by 300 μm (NeuroNexus, Inc.). Neuronal signals were sampled at 30 kHz (Scout Processor; Ripple, Inc.). Electrodes within a tetrode were separated by 25 μm and arranged in a diamond pattern. Because the amplitude of a recorded action potential depends on the distance between the neuron and each contact, tetrodes provide more distinct waveforms for distinguishing between neurons (52). Spike sorting was performed offline with the KlustaKwik semiautomatic clustering algorithm in MClust-4.0 followed by manual refinement based on waveform profile, stability, and refractory period violations with Offline Sorter (Plexon, Inc.). Only well-isolated single neurons verified by at least two authors (V3A: *N* = 692; CIP: *N* = 437) were included.

### Data Analyses

The visual feature selectivity, choice-related activity, and saccade-related activity of the V3A and CIP neurons analyzed here have been extensively compared (35, 36). As such, the present analyses test for functional clustering within each of these domains, cross-area differences in clustering, and whether the strength of clustering was moderated by mixed selectivity.

#### Quantification of visual feature selectivity.

Visual responses were calculated from the median visual response latency (V3A: 46 ms; CIP: 52 ms) to the start of choice-related activity (V3A: 191 ms; CIP: 202 ms) for each population. Within these windows, individual neuron responses reflect visual selectivity without detectable choice signals (see results) (35). Except where indicated, responses were baseline subtracted. At each distance, the statistical significance of 3-D orientation tuning was tested with an ANOVA (*P* < 0.05; Bonferroni–Holm corrected for *N* = 4 distances). Significant tuning curves were fit with a Bingham function (32, 53):

(*1*) $$\text{R}\left(x\right)=\text{DC}+\text{G}\cdot \exp(-\lambda_{2})\cdot \exp\left[{\text{λ}}_{1}{\left({\text{μ}}_{1}^{\text{T}}x\right)}^{2}+{\text{λ}}_{2}{\left({\text{μ}}_{2}^{\text{T}}x\right)}^{2}\right]$$

Here, *x* is a unit vector corresponding to a given orientation, DC is an offset, G is the response gain, λ_1_ sets the anisotropy of the tuning curve, and λ_2_ sets the tuning bandwidth. The term exp(−λ_2_) is a regularization parameter that ensures that G sets the gain independently of the bandwidth. The orthonormal vectors µ_1_ and µ_2_ are

(*2*) $$\begin{array}{c} \mu_{1}=\;-\;\left[\begin{array}{c} \sin\left(\phi \right)\cdot \cos\left(s*\right)\cdot \cos\left(t*\right)+\cos\left(\phi \right)\cdot \sin\left(t*\right)\\ \sin\left(\phi \right)\cdot \cos\left(s*\right)\cdot \sin\left(t*\right)-\cos\left(\phi \right)\cdot \cos\left(t*\right)\\ -\sin\left(\phi \right)\cdot \sin\left(s{*}^{*}\right) \end{array}\right]\\ {\text{μ}}_{2}=\left[\begin{array}{c} \sin\left(s*\right)\cdot \cos\left(t*\right)\\ \sin\left(s*\right)\cdot \sin\left(t*\right)\\ \cos\left(s*\right) \end{array}\right] \end{array}$$

Here, t* is the preferred tilt, s* is the preferred slant, and ϕ sets the axis of anisotropy by rotating the tuning curve about the preferred slant-tilt.

The preferred distance was defined as the weighted mean of the four distances, where each weight was the maximum firing rate (not baseline subtracted) across the corresponding orientation tuning curve:

(*3*) $$D_{\text{pref}}=\frac{{\text{ΣR}}_{i}D_{i}}{{\text{ΣR}}_{i}}$$

Here, *D_i_* is the *i*th distance, and R*_i_* is the maximum firing rate at that distance.

Selectivity for lower-level visual features versus higher-level 3-D object pose was quantified on a continuum by fitting the joint orientation and distance tuning curves with a separable model (24, 35, 36):

(*4*) R(θ,D)=DC+G·H(θ)·F(D)

Here, θ is the orientation (slant and tilt), *D* is the distance, DC is an offset, G is the response gain, *H*(θ) is the orientation tuning curve, and *F*(*D*) is the distance tuning curve.

A tolerance index describing how much the shape of the orientation tuning curve depended on the distance was then calculated by taking the average of the Pearson correlations between the observed and fitted orientation tuning curves at each distance (35, 36). Values closer to 0 indicate that the shape heavily depended on distance (implying lower-level feature selectivity). Values closer to 1 indicate that the shape was more invariant to distance (implying 3-D pose tuning).

#### Quantification of choice- and saccade-related activity.

Choice tuning was measured from the start of choice-related activity within each area (V3A: 191 ms; CIP: 202 ms) to the end of the stimulus presentation, using frontoparallel plane trials only (35, 36). To increase statistical power, responses were pooled across distance after *z* scoring of all frontoparallel plane responses at each distance. Saccade tuning was measured from the start of saccade-related activity within each area (V3A: −108 ms; CIP: −102 ms) to the saccade onset. Responses were baseline subtracted.

Significant choice and saccade direction tuning curves (ANOVA, *P* < 0.05) were fit with a von Mises function:

(*5*) $$\text{R}\left(\theta \right)=\text{DC}+\text{G}\cdot \text{exp}(-\kappa )\cdot \text{exp}\left[\kappa \cdot \cos\left(\theta -\theta_{0}\right)\right]$$

Here, θ is the choice or saccade direction, DC is an offset, G is the response gain, κ sets the bandwidth, and θ_0_ is the preference. The term exp(−κ) is a regularization parameter that ensures that G sets the gain independently of the bandwidth.

#### Discrimination indices.

To quantify how well the responses of single neurons to preferred and nonpreferred conditions could be discriminated relative to their response variability, we computed pose, choice, and saccade discrimination indexes (PDI, CDI, and SDI, respectively) (34–36, 54):

(*6*) $$\mathrm{DI}=\frac{R_{\text{max}}-R_{\text{min}}}{R_{\text{max}}-R_{\text{min}}+2\cdot \sqrt{\frac{\text{SSE}}{\left(N\;-M\right)}}}$$

Here, *R*_max_ and *R*_min_ are maximum and minimum mean responses across the tuning curve, SSE is the sum squared error around the mean response to each condition, *N* is the total number of trials, and *M* is the number of conditions (PDI: *M* = 132; CDI: *M* = 8; SDI: *M* = 8). Values closer to 0 indicate weaker discriminability, whereas values closer to 1 indicate greater discriminability.

The DI reflects the difference in mean firing rates to preferred and nonpreferred conditions as well as the response variability. As such, they were correlated but distinguishable from *R*_max_ – *R*_min_ [Spearman ρ; PDI: V3A = 0.66, CIP = 0.52; CDI: V3A > 0.99, CIP > 0.99 (the especially high correlations for the CDI are a consequence of the *z* scoring); SDI: V3A = 0.80, CIP = 0.69].

#### Statistical analyses.

Neuronal pairs were defined as two neurons with significant tuning simultaneously recorded on the same tetrode. For each pair, we quantified the similarity of their responses by calculating the Pearson correlation (*r*) between the tuning curves as well as taking absolute differences between fitted parameter values and DIs.

Differences in 3-D orientation tuning (which was measured at 4 distances) were calculated by defining vectors of each property. Each element was the parameter value from the Bingham fit at the corresponding distance. Using only distances at which both neurons in a pair had significant tuning, we took the root mean square error (RMSE) between the vectors. Absolute differences between individual parameter values and RMSEs between vectors of parameter values are both indicated with the notation |Δ·|.

The statistical significance of clustering was determined with permutation tests (8). For each permutation, we shuffled the spatial location of all included neurons (i.e., significantly tuned neurons from tetrodes with >1 tuned neuron) from the same area and hemisphere of the monkey. For each permuted pair, we quantified the similarity of each property. Median values were then taken across pairs. This procedure was repeated 10,000 times to generate null distributions. The *P* values were calculated as the proportion of null distribution values that were smaller (or larger for correlations) than the median value from the unpermuted data. Exact *P* values are reported unless the unpermuted median was smaller (or larger) than the minimum (or maximum) of the null distribution, in which case we report *P* < 1 × 10^−4^. To verify that the findings were robust to the analysis method, we also performed shuffling tests (55). The significance of the results differed in only 2 out of 32 tests. Both instances related to visual selectivity in V3A. Specifically, the clustering of tuning isotropy (λ_1_) was not significant with a permutation test but was significant with a shuffling test. The clustering of tuning bandwidth (λ_2_) was significant with a permutation test but not a shuffling test. Because the differences were in opposite directions, the choice in statistical test did not affect the overall conclusions. Statistics were Bonferroni–Holm corrected for multiple comparisons according to the number of comparisons within each domain: visual (*N* = 8), choice (*N* = 4), and saccade (*N* = 4).

To test for cross-area differences in the strength of clustering, we used Wilcoxon’s rank-sum test to compare distributions of tuning curve correlations across pairs. We also contrasted the number of properties that clustered in each area. Importantly, we did not perform cross-area comparisons of absolute differences or RMSEs between parameter values. Such comparisons would have been problematic because the upper bound of differences in an area depends on the underlying distribution of values. For illustration, consider the extreme scenario in which all neurons prefer the same stimulus. In that case, the distribution of preference differences consists of zeros only and conclusions regarding clustering would be trivial. More generally, distributions of parameter differences will depend on other descriptive statistics (variance, skew, etc.) of the underlying distribution of values. This makes cross-area comparisons of parameter differences susceptible to inferential errors (see discussion).

To test whether the strength of clustering depended on whether the neurons showed mixed selectivity, we used a linear mixed-effects model. Specifically, the model tested whether the similarity of properties within each domain depended on whether neither, one, or both neurons in a pair were tuned in the other domains:

(*7*) $$\Delta ∼{\text{b}}_{0}+{\text{b}}_{1}{\text{M}}_{1}+{\text{b}}_{2}{\text{M}}_{2}+{\text{b}}_{3}{\text{M}}_{1}\cdot {\text{M}}_{2}$$

Here, Δ is either the absolute difference (or RMSE) of the analyzed property or the tuning curve correlation, and M_1_ and M_2_ indicate selectivity in the other domains, with 0, 1, or 2 denoting the number of neurons in the pair with significant tuning. For example, the model that tested whether the similarity of saccade direction preferences (|Δθ_S_|) depended on whether the neurons had visual (V) and/or choice (C) tuning was |Δθ_S_| ∼ b_0_ + b_1_V + b_2_C + b_3_V · C.

## RESULTS

To assess the clustering of visual feature selectivity, choice-related activity, and saccade-related activity in V3A and CIP, we used correlational and parametric methods to quantify the similarity of well-isolated single neurons’ spiking activity.

### Clustering of Visual Feature Selectivity

**Figure 1.**
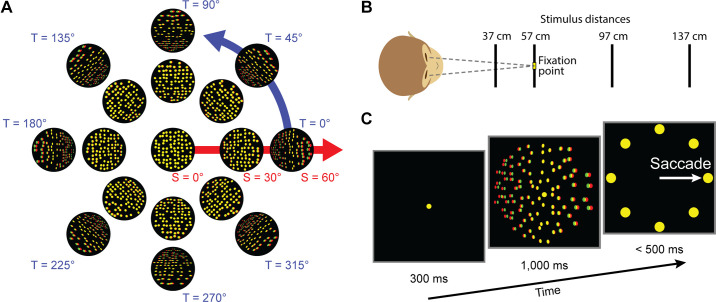
Visual stimuli and tilt discrimination task. *A*: slant-tilt coordinates for planar surface orientation. Tilt (T) specifies the direction that the plane is oriented in depth. Slant (S) specifies how much it is oriented in depth. Dots were rendered with perspective and stereoscopic cues (shown here as red-green anaglyphs). *B*: planes were presented at 4 distances with fixation at 57 cm. *C*: tilt discrimination task. A central target was fixated for 300 ms. A plane then appeared for 1,000 ms while fixation was maintained. The fixation target and plane then disappeared, and 8 choice targets appeared. A saccade was made to the target at the perceived nearest side of the plane.

Visual feature selectivity was measured by presenting 3-D oriented planar surfaces (Fig. 1*A*) at four distances (Fig. 1*B*). The monkeys simultaneously performed an eight-alternative forced-choice tilt discrimination task (Fig. 1*C*). Visually responsive neurons were identified by performing a one-way ANOVA over orientation at each distance (*P* < 0.05; Bonferroni–Holm corrected, *N* = 4). If at least one distance showed significant tuning, the neuron was classified as visually responsive (V3A: 549/692, 79%; CIP: 363/437, 83%). To assess the clustering of visual feature selectivity, we identified all tetrode recordings with at least two tuned neurons. Across those recordings, the average number of visually responsive neurons was 2.4 in both V3A (range: 2 to 4) and CIP (range: 2 to 5). In V3A, there was a total of 271 neuronal pairs from 364 neurons on 154 tetrodes (mean: 1.76 pairs/tetrode). In CIP, there were 175 pairs from 226 neurons on 95 tetrodes (mean: 1.84 pairs/tetrode).

**Figure 2.**
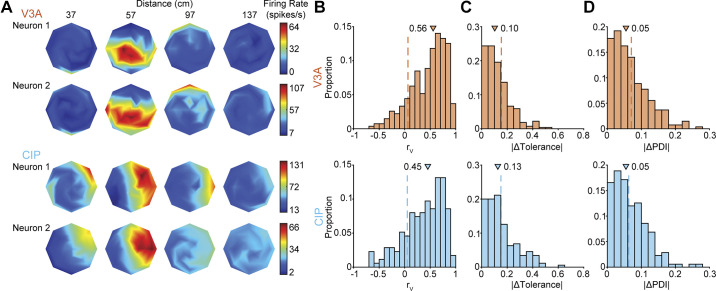
Clustering of 3-dimensional (3-D) pose tuning. *A*: example tuning curves of neuronal pairs from V3A [Pearson correlation (*r*_V_) = 0.91 and absolute differences in tolerance of 3-D orientation tuning curve shape to distance (|ΔTolerance|) = 0.12, pose discrimination index (|ΔPDI|) = 0.02, orientation preference (|Δθ_V_|) = 15°, tuning bandwidth (|Δλ_2_|) = 1.21, tuning anisotropy (|Δλ_1_|) = 3.20, axis of tuning anisotropy (|Δϕ|) = 30°, and distance preferences (|Δ*D*|) = 4 cm] and caudal intraparietal area (CIP) (*r*_V_ = 0.80, |ΔTolerance| = 0.03, |ΔPDI| = 0.05, |Δθ_V_| = 17°, |Δλ_2_| = 0.78, |Δλ_1_| = 0.59, |Δϕ| = 18°, |Δ*D*| = 13 cm). Heat maps show 3-D orientation tuning at each distance, plotted in slant-tilt coordinates (Fig. 1*A*). *B*: *r*_V_ between tuning curves of neuronal pairs in V3A (*top*, orange) and CIP (*bottom*, blue). *C*: |ΔTolerance|. *D*: |ΔPDI|. In *B–D*, triangles mark median values. Dashed vertical lines mark median values obtained by chance.

As the first test of whether visual feature selectivity functionally clustered, we compared the 3-D pose (i.e., joint orientation and distance) tuning curves of each neuronal pair. Example tuning curves are shown for a neuronal pair from each area in Fig. 2*A*. To quantify the similarity of the tuning curves, we calculated their Pearson correlation. The distributions of correlation coefficients (*r*_V_) are shown in Fig. 2*B* [V3A: median = 0.56, interquartile range (IQR) = 0.49; CIP: median = 0.45, IQR = 0.55]. In both areas, the median correlation was significantly larger than expected by chance (*P* < 1 × 10^−4^; Table 1, first row), indicating that visual selectivity clustered in V3A and CIP. However, the median correlation was significantly larger in V3A than CIP (*P* = 1.6 × 10^−2^), suggesting that the clustering was stronger in V3A.

**Table 1.** Clustering of visual feature selectivity

Response Property	*P* Value	
	V3A	CIP
Tuning correlation (*r*_V_)	**<1 × 10^−4^**	**<1 × 10^−4^**
Tolerance index	**<1 × 10^−4^**	2.4 × 10^−2^
Pose discrimination index (PDI)	**3 × 10^−4^**	4.7 × 10^−2^
Orientation preference (θ_V_)	**<1 × 10^−4^**	**<1 × 10^−4^**
Tuning bandwidth (λ_2_)	**8.9 × 10^−3^**	0.17
Tuning anisotropy (λ_1_)	0.33	0.28
Axis of anisotropy (ϕ)	**<1 × 10^−4^**	0.38
Distance preference (*D*)	**1× 10^−4^**	5.3 × 10^−2^

Significant *P* values indicating clustering are in bold (Bonferroni–Holm corrected, *N* = 8 comparisons). CIP, caudal intraparietal area.

For neurons that encode 3-D object pose, the joint tuning for orientation and distance should be multiplicatively separable (24). Consistent with 3-D pose representations being hierarchically computed, the separability of pose tuning increases between V3A and CIP (35, 36), as reflected in cross-area differences in a tolerance index that quantifies how much the 3-D orientation tuning curve shape depends on distance (materials and methods, *Eq. 4*). To test whether selectivity for lower-level visual features (low tolerance values) versus higher-level 3-D object pose (high tolerance values) clustered, we took the absolute difference in tolerance values for each pair (|ΔTolerance|). The distributions of |ΔTolerance| are shown in Fig. 2*C* (V3A: median = 0.10, IQR = 0.13; CIP: median = 0.13, IQR = 0.14). Further supporting that visual selectivity was more strongly clustered in V3A, the tolerance index clustered in V3A (*P* < 1 × 10^−4^) but not CIP (*P* = 2.4 × 10^−2^; not significant after Bonferroni–Holm correction; Table 1, second row).

To test whether the strength of visual selectivity clustered, we calculated a pose discrimination index for each neuron (PDI; materials and methods, *Eq. 6*). For each pair, we then took the absolute difference between the PDI values (|ΔPDI|). The distributions of |ΔPDI| are shown in Fig. 2*D* (V3A: median = 0.05, IQR = 0.07; CIP: median = 0.05, IQR = 0.07; same values due to rounding). Although the median differences in PDI values were nearly identical, the strength of selectivity significantly clustered in V3A (*P* = 3 × 10^−4^) but not CIP (*P* = 4.7 × 10^−2^; not significant after Bonferroni–Holm correction; Table 1, third row). This again supports stronger clustering of visual selectivity in V3A and highlights that clustering measures based on differences in selectivity must be interpreted relative to the underlying distribution of values within the area (see materials and methods and discussion).

**Figure 3.**
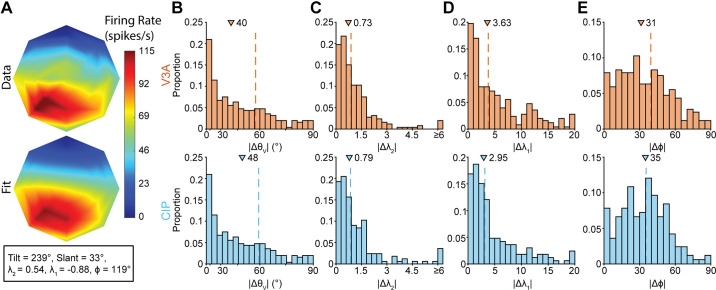
Clustering of 3-dimensional (3-D) orientation tuning. *A*: example tuning curve from V3A (*top*) with Bingham fit (*r* = 0.96; *bottom*). *Inset*, the Bingham parameter values. *B*: absolute differences in the orientation preferences of neuronal pairs (|Δθ_V_|) in V3A (*top*, orange) and caudal intraparietal area (CIP) (*bottom*, blue), plotted over an equal area axis. *C*: absolute differences in tuning bandwidths (|Δλ_2_|). *D*: absolute differences in tuning anisotropies (|Δλ_1_|). *E*: absolute differences in the axes of tuning anisotropy (|Δϕ|). In *B–E*, triangles mark median values. Dashed vertical lines mark median values obtained by chance.

**Figure 4.**
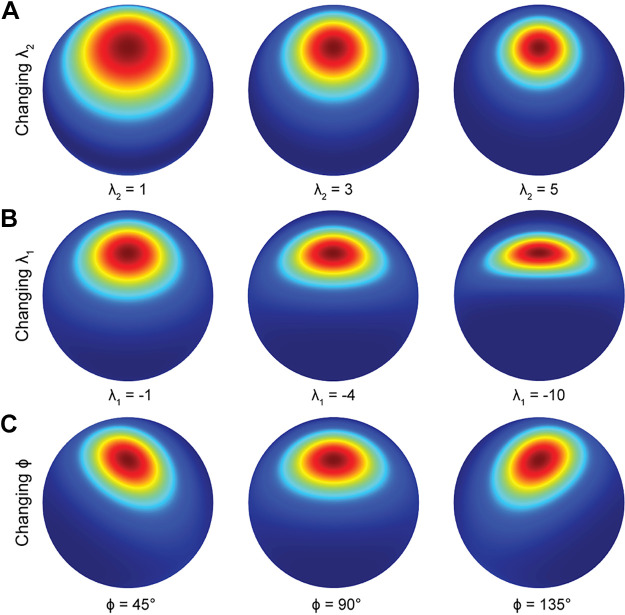
Bingham parameters. To illustrate how orientation tuning depends on parameters λ_2_ (tuning bandwidth), λ_1_ (tuning anisotropy), and ϕ (axis of tuning anisotropy), tuning curves were modeled with a preferred tilt of 90° and slant of 30°. *A*: changing λ_2_ with λ_1_ = 0. *B*: changing λ_1_ with λ_2_ = 3 and ϕ = 90°. *C*: changing ϕ with λ_1_ = −2.5 and λ_2_ = 3.

We next examined the clustering of orientation selectivity by performing parametric comparisons of Bingham function fits to the orientation tuning curves (materials and methods, *Eqs. 1* and *2*) (32, 34–36). An example orientation tuning curve measured at 57 cm along with its Bingham function fit is shown for a V3A neuron in Fig. 3*A*. Using the fitted parameter values, we compared four properties: orientation preference (θ), tuning bandwidth (λ_2_), tuning anisotropy (λ_1_), and the axis of anisotropy about the preferred slant-tilt (ϕ). Schematics illustrating how orientation tuning depends on these parameters are shown in Fig. 4.

To quantify the clustering of orientation preference, we computed for each pair the RMSE between preferred orientations at distances that both neurons were tuned (|Δθ_V_|) (materials and methods). As such, only pairs with at least one distance at which both neurons were tuned were included (V3A: 253/271 pairs; CIP: 166/175 pairs). The distributions of |Δθ_V_| are shown in Fig. 3*B* (V3A: median = 40°, IQR = 37°; CIP: median = 48°, IQR = 36°). In both areas, the median |Δθ_V_| was significantly smaller than expected by chance (*P* < 1 × 10^−4^; Table 1, fourth row), indicating that orientation preference clustered in V3A and CIP.

We next tested whether tuning bandwidth clustered by computing for each pair the RMSE between λ_2_ values at distances that both neurons were tuned (|Δλ_2_|). The distributions of |Δλ_2_| are shown in Fig. 3*C* (V3A: median = 0.73, IQR = 1.06; CIP: median = 0.79, IQR = 1.20). Further supporting that visual selectivity was more strongly clustered in V3A, tuning bandwidth significantly clustered in V3A (*P* = 8.9 × 10^−3^) but not CIP (*P* = 0.17; Table 1, fifth row).

Finally, we tested whether the amount of anisotropy in the orientation tuning curve (λ_1_) and axis about which anisotropies occurred (ϕ) clustered by calculating corresponding RMSEs (|Δλ_1_| and |Δϕ|, respectively). The distributions of |Δλ_1_| are shown in Fig. 3*D* (V3A: median = 3.63, IQR = 6.47; CIP: median = 2.95, IQR = 5.08). Tuning anisotropy was not clustered in either area (*P* ≥ 0.28; Table 1, sixth row). The distributions of |Δϕ| are shown in Fig. 3*E* (V3A: median = 31°, IQR = 32°; CIP: median = 35°, IQR = 27°). In this case, there was clustering in V3A (*P* < 1 × 10^−4^) but not CIP (*P* = 0.38; Table 1, seventh row), indicating that whereas the amount of anisotropy did not cluster in either area, the axis about which anisotropies occurred did cluster in V3A.

**Figure 5.**
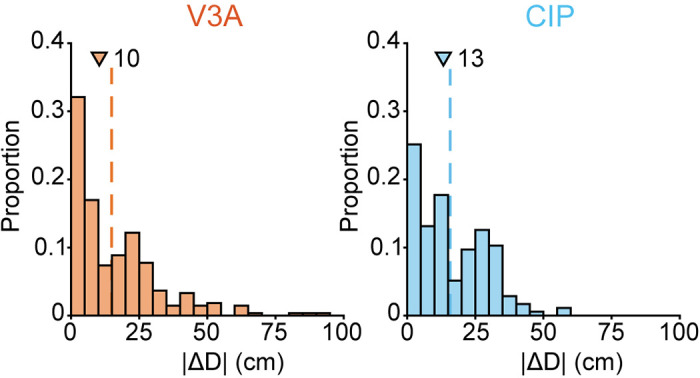
Clustering of preferred distance. Absolute differences in the distance preferences of neuronal pairs (|Δ*D*|) in V3A (*left*, orange) and caudal intraparietal area (CIP) (*right*, blue). Triangles mark median values. Dashed vertical lines mark median values obtained by chance.

As the final test of whether visual feature selectivity clustered, we compared the distance preferences of each pair. The preferred distance (*D*_pref_) of each neuron was estimated by calculating the weighted average of stimulus distances with each distance weighted by the maximum firing rate across the corresponding orientation tuning curve (materials and methods, *Eq. 3*). For each pair, we then took the absolute difference between the preferred distances (|Δ*D*|). The distributions of |Δ*D*| are shown in Fig. 5 (V3A: median = 10 cm, IQR = 22 cm; CIP: median = 13 cm, IQR = 22 cm). The median |Δ*D*| was significantly smaller than expected by chance in V3A (*P* = 1 × 10^−4^) but not CIP (*P* = 5.3 × 10^−2^; Table 1, eighth row), further supporting that visual feature selectivity was more strongly clustered in V3A.

The above analyses indicate that visual feature selectivity clustered in both areas. However, the tuning curves of neuronal pairs were more strongly correlated in V3A. In addition, substantially more properties clustered in V3A (7/8 comparisons) than in CIP (2/8 comparisons, with clustering attributable to the local similarity of orientation preferences only). These findings collectively imply that visual selectivity was more strongly clustered in V3A than in CIP.

### Clustering of Choice-Related Activity

**Figure 6.**
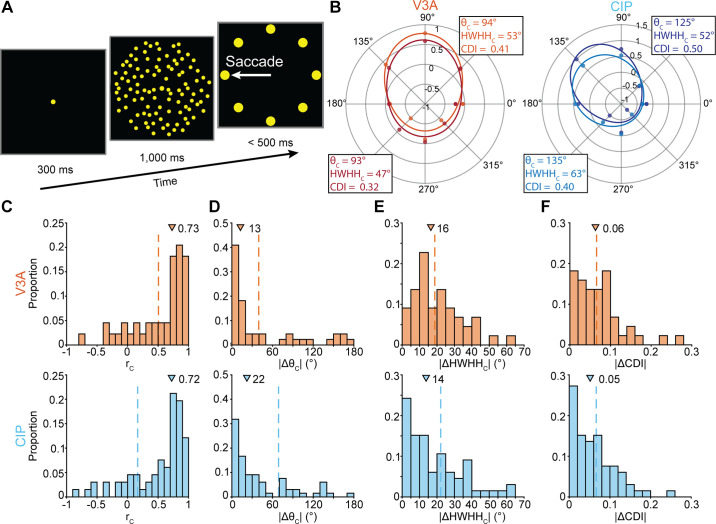
Clustering of choice-related activity. *A*: choice activity was measured during the presentation of frontoparallel planes, which were task ambiguous (cf. Fig. 1*C*). *B*: example tuning curves of neuronal pairs from V3A [*left*; Pearson correlation (*r*_C_) = 0.91 and absolute differences in choice preference (|Δθ_C_|) = 1°, half-width at half-height (|ΔHWHH_C_|) = 6°, and choice discrimination index |ΔCDI| = 0.09] and caudal intraparietal area (CIP) (*right*; *r*_C_ = 0.97, |Δθ_C_| = 10°, |ΔHWHH_C_| = 11°, |ΔCDI| = 0.10). Colors correspond to different neurons. Data points are mean *z*-scored responses, and curves are von Mises fits. *Insets*, θ_C_, HWHH_C_, and CDI. *C*: *r*_C_ between tuning curves of neuronal pairs in V3A (*top*, orange) and CIP (*bottom*, blue). *D*: |Δθ_C_|. *E*: |ΔHWHH_C_|. *F*: |ΔCDI|. In *C–F*, triangles mark median values. Dashed vertical lines mark median values obtained by chance.

We next identified neurons that carried choice-related activity during the tilt discrimination task by testing whether their responses to frontoparallel planes (which were task ambiguous) covaried with the monkey’s choices (ANOVA, *P* < 0.05; Fig. 6*A*). Across the populations, 25% (172/692) of the V3A and 46% (201/437) of the CIP neurons had significant choice tuning that began in V3A 191 ms after stimulus onset and in CIP 202 ms after (35, 36). As a control, we confirmed that before these time points the prevalence of choice activity was consistent with the expected rate of false positives (V3A: 7%, 46/692; CIP: 5%, 22/437). That activity was also not associated with whether the neurons were classified as carrying choice-related activity, since only 16 of the 46 V3A and 13 of the 22 CIP neurons had choice tuning during the choice analysis window (35). To assess the clustering of choice-related activity, we identified all tetrode recordings with at least two tuned neurons. Across those recordings, the average number of neurons with choice-related activity was 2.2 (range: 2 to 4) in both areas. In V3A, there was a total of 44 pairs from 68 neurons on 31 tetrodes (mean: 1.42 pairs/tetrode). In CIP, there were 66 pairs from 103 neurons on 47 tetrodes (mean: 1.40 pairs/tetrode).

Example choice tuning curves are shown for a neuronal pair from each area in Fig. 6*B*. To quantify the similarity of the tuning curves, we calculated their Pearson correlation. The distributions of correlation coefficients (*r*_C_) are shown in Fig. 6*C* (V3A: median = 0.73, IQR = 0.57; CIP: median = 0.72, IQR = 0.57). In both areas, the median correlation was significantly larger than expected by chance (*P* ≤ 3 × 10^−4^; Table 2, first row), indicating that choice-related activity clustered in V3A and CIP. The median correlations were not significantly different across the areas (*P* = 0.57). Thus, choice-related activity was substantially more prevalent in CIP than V3A, but the local similarity of choice tuning (when present) was similar in the two areas.

**Table 2.** Clustering of choice-related activity

Response Property	*P* Value	
	V3A	CIP
Tuning correlation (*r*_C_)	**3 × 10^−4^**	**<1 × 10^−4^**
Choice preference (θ_C_)	**<1 × 10^−4^**	**<1 × 10^−4^**
Tuning bandwidth (HWHH_C_)	0.25	**2.7 × 10^−3^**
Choice discrimination index (CDI)	0.37	**4.5 × 10^−2^**

Significant *P* values indicating clustering are in bold (Bonferroni–Holm corrected, *N* = 4 comparisons). CIP, caudal intraparietal area.

To determine which features of the choice-related activity clustered, we fit each tuning curve with a von Mises function (materials and methods, *Eq. 5*; Fig. 6*B*, solid curves). We then quantified the clustering of choice preferences (θ_C_) by taking the absolute difference between the preferences of each pair (|Δθ_C_|). The distributions of |Δθ_C_| are shown in Fig. 6*D* (V3A: median = 13°, IQR = 77°; CIP: median = 22°, IQR = 65°). The median |Δθ_C_| was significantly smaller than expected by chance in both areas (*P* < 1 × 10^−4^; Table 2, second row), indicating that choice preferences clustered in V3A and CIP. We likewise assessed the clustering of tuning bandwidths (half-width at half-height; HWHH_C_) by taking their absolute difference for each pair (|ΔHWHH_C_|). The distributions of |ΔHWHH_C_| are shown in Fig. 6*E* (V3A: median = 16°, IQR = 16°; CIP: median = 14°, IQR = 23°). Choice tuning bandwidth was not significantly clustered in V3A (*P* = 0.25) but was clustered in CIP (*P* = 2.7 × 10^−3^; Table 2, third row), suggesting that choice-related activity was more strongly clustered in CIP.

Finally, we tested whether the strength of choice selectivity clustered by calculating a choice discrimination index for each neuron (CDI; materials and methods, *Eq. 6*). For each pair, we then took the absolute difference between the CDI values (|ΔCDI|). The distributions of |ΔCDI| are shown in Fig. 6*F* (V3A: median = 0.06, IQR = 0.06; CIP: median = 0.05, IQR = 0.07). The strength of choice selectivity was not significantly clustered in V3A (*P* = 0.37) but was clustered in CIP (*P* = 4.5 × 10^−2^; Table 2, fourth row), supporting that clustering was stronger in CIP.

The above analyses indicate that choice-related activity clustered in both areas. However, more properties clustered in CIP (4/4 comparisons) than in V3A (2/4 comparisons, with clustering attributable to the local similarity of choice preferences only). Although visual and choice preferences tend to align in both areas (35, 36), the present findings reveal stronger clustering of visual selectivity in V3A and stronger clustering of choice activity in CIP. This may reflect differences in the areas’ computational roles, with V3A having a greater role in visual signal processing and CIP having a greater role in 3-D perceptual decision processes.

### Clustering of Saccade-Related Activity

**Figure 7.**
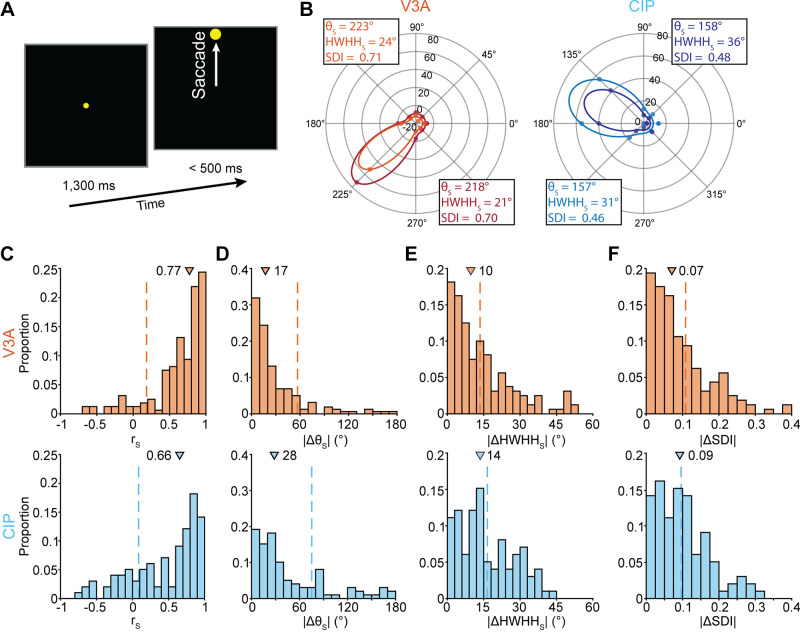
Clustering of saccade-related activity. *A*: visually guided saccade task. A central target was fixated for 1,300 ms. The fixation target then disappeared, and a saccade target appeared at 1 of 8 locations. A saccade was then made to that target. *B*: example tuning curves of neuronal pairs from V3A [*left*; Pearson correlation (*r*_S_) = 0.99 and absolute differences in saccade direction preferences (|Δθ_S_|) = 5°, half-width at half-height (|ΔHWHH_S_|) = 3°, and saccade discrimination index (|ΔSDI|) = 0.01] and caudal intraparietal area (CIP) (*right*; *r*_S_ = 0.97, |Δθ_S_|= 1°, |ΔHWHH_S_| = 5°, |ΔSDI| = 0.02). Colors correspond to different neurons. Data points are mean responses, and curves are von Mises fits. *Insets*, θ_S_, HWHH_S_, and SDI. *C*: *r*_S_ between tuning curves of neuronal pairs in V3A (*top*, orange) and CIP (*bottom*, blue). *D*: |Δθ_S_|. *E*: |ΔHWHH_S_|. *F*: |ΔSDI|. In *C–F*, triangles mark median values. Dashed vertical lines mark median values obtained by chance.

We further identified neurons that carried saccade-related activity during a visually guided saccade task by testing whether the activity depended on the saccade direction (ANOVA, *P* < 0.05; Fig. 7*A*). Across the populations, 60% (415/692) of V3A and 63% (274/437) of CIP neurons carried saccade-related activity that predicted the direction and timing of eye movements (35, 36). That activity began in V3A 108 ms before the saccade and in CIP 102 ms before. Importantly, the saccade-related activity was functionally distinct from the choice-related activity (35, 36). To assess the clustering of saccade-related activity, we identified all tetrode recordings with at least two tuned neurons. Across those recordings, the average number of neurons with saccade-related activity was 2.3 (range: 2 to 4) in both areas. In V3A, there were 160 pairs from 224 neurons on 97 tetrodes (mean: 1.65 pairs/tetrode). In CIP, there were 99 pairs from 152 neurons on 67 tetrodes (mean: 1.48 pairs/tetrode).

Example saccade direction tuning curves are shown for a neuronal pair from each area in Fig. 7*B*. To quantify the similarity of the tuning curves, we calculated their Pearson correlation. The distributions of correlation coefficients (*r*_S_) are shown in Fig. 7*C* (V3A: median = 0.77, IQR = 0.38; CIP: median = 0.66, IQR = 0.72). In both areas, the median correlation was significantly larger than expected by chance (*P* < 1 × 10^−4^; Table 3, first row), indicating that saccade-related activity clustered in V3A and CIP. However, the median correlation was significantly larger in V3A than CIP (*P* = 1.8 × 10^−3^), suggesting that the clustering was stronger in V3A.

**Table 3.** Clustering of saccade-related activity

Response Property	*P* Value	
	V3A	CIP
Tuning correlation (*r*_S_)	**<1 × 10^−4^**	**<1 × 10^−4^**
Saccade direction preference (θ_S_)	**<1 × 10^−4^**	**<1 × 10^−4^**
Tuning bandwidth (HWHH_S_)	**7 × 10^−4^**	3.3 × 10^−2^
Saccade discrimination index (SDI)	**<1 × 10^−4^**	0.40

Significant *P* values indicating clustering are in bold (Bonferroni-Holm corrected, *N* = 4 comparisons). CIP, caudal intraparietal area.

To determine which features of the saccade-related activity clustered, we fit each saccade direction tuning curve with a von Mises function (materials and methods, *Eq. 5*; Fig. 7*B*, solid curves). We then quantified the clustering of saccade direction preferences (θ_S_) by taking the absolute difference between the preferences of each pair (|Δθ_S_|). The distributions of |Δθ_S_| are shown in Fig. 7*D* (V3A: median = 17°, IQR = 30°; CIP: median = 28°, IQR = 60°). The median |Δθ_S_| was significantly smaller than expected by chance in both areas (*P* < 1 × 10^−4^; Table 3, second row), indicating that the direction preferences clustered in V3A and CIP. We likewise assessed the clustering of tuning bandwidths (HWHH_S_) by taking their absolute difference for each pair (|ΔHWHH_S_|). The distributions of |ΔHWHH_S_| are shown in Fig. 7*E* (V3A: median = 10°, IQR = 17°; CIP: median = 14°, IQR = 18°). Saccade direction tuning bandwidth was significantly clustered in V3A (*P* = 7 × 10^−4^) but not CIP (*P* = 3.3 × 10^−2^, not significant after Bonferroni–Holm correction; Table 3, third row), supporting that clustering was stronger in V3A.

Finally, we tested whether the strength of saccade direction selectivity clustered by calculating a saccade discrimination index for each neuron (SDI; materials and methods, *Eq. 6*). For each pair, we then took the absolute difference between the SDI values (|ΔSDI|). The distributions of |ΔSDI| are shown in Fig. 7*F* (V3A: median = 0.07, IQR = 0.10; CIP: median = 0.09, IQR = 0.10). The strength of saccade direction selectivity was significantly clustered in V3A (*P* < 1 × 10^−4^) but not CIP (*P* = 0.40; Table 3, fourth row), further supporting that clustering was stronger in V3A.

The above analyses indicate that saccade-related activity clustered in both areas. However, the tuning curves of neuronal pairs were more strongly correlated in V3A. In addition, more properties clustered in V3A (4/4 comparisons) than in CIP (2/4 comparisons, with clustering attributable to the local similarity of saccade direction preferences only). Stronger clustering of visual selectivity and saccade-related activity in V3A than in CIP may reflect a greater role for V3A in the parallel processing of visual and oculomotor-related signals. In contrast, stronger clustering of choice-related activity in CIP than in V3A may reflect a greater role for CIP in 3-D perceptual decision processes and the synthesis of visual and oculomotor signals (35, 36).

### Clustering Had Little to No Dependence on Mixed Selectivity

Having assessed the clustering of visual and choice- and saccade-related activity, we finally tested whether the strength of clustering depended on whether the neurons showed mixed selectivity. We first compared the prevalence of unimodal and mixed selectivity across the two areas. As indicated above, nearly twice as many CIP (46%) as V3A (25%) neurons carried choice-related activity (35, 36). To further compare the prevalence of mixed visual and saccade signals, we marginalized over the presence/absence of choice activity. Across the V3A neurons recorded on tetrodes with more than one neuron (*N* = 493), 58 (12%) showed neither visual selectivity nor saccade activity, 151 (31%) showed visual selectivity only, 40 (8%) showed saccade activity only, and 244 (49%) showed both visual and saccade activity. A similar pattern was observed across the corresponding 292 CIP neurons: 24 (8%) showed neither visual selectivity nor saccade activity, 85 (29%) showed visual selectivity only, 25 (9%) showed saccade activity only, and 158 (54%) showed both visual and saccade activity. Thus, the prevalence of visual selectivity only, saccade-related activity only, and mixed selectivity was similar in the two areas.

We finally tested whether the strength of clustering within each domain depended on whether the neurons showed selectivity in the other domains. For each examined property (32 total: 8 visual, 4 choice, and 4 saccadic from 2 areas), we used a linear mixed-effects model to test whether the similarity between neuronal pairs depended on whether neither, one, or both neurons were tuned in the other domains (*Eq. 7*). For 31/32 comparisons, there was no significant relationship (*P* ≥ 0.11). The only significant case was that saccade direction preferences in CIP tended to be more similar if both neurons showed choice tuning (b_1_ = −24.9, *P* = 4.5 × 10^−3^). These results indicate that mixed selectivity had little to no bearing on within-domain functional clustering.

## DISCUSSION

In this study, we assessed whether the visual feature selectivity, choice-related activity, and saccade-related activity of neurons in macaque areas V3A and CIP functionally cluster. In both areas, we found statistically significant clustering of tuning preferences in all three domains. However, there were also domain-specific, cross-area differences in the clustering of other properties such as bandwidth, and therefore the overall strength of clustering. Area V3A showed stronger clustering of visual selectivity and saccade-related activity. Area CIP showed stronger clustering of choice-related activity. The clustering of neurons with similar functional properties is thought to facilitate computations within the clustered feature space (1–7, 22). As such, cross-area differences in clustering may provide insights into the functional roles of the areas. Stronger clustering of visual and saccade signals in V3A may reflect wiring patterns that are optimized for within-domain (visual or oculomotor, though often multiplexed) processing that supports the subsequent computation of invariant object representations and sensorimotor transformations. Consistent with this possibility, V3A neurons that carry saccade-related activity are less selective for high-level 3-D visual features than those without saccade-related activity (36). Stronger clustering of choice-related activity in CIP may likewise reflect wiring that is optimized to synthesize those visual and oculomotor signals. Given the relative sparsity of V3A pairs for which both neurons carried choice activity, some caution is required in concluding cross-area differences based on that finding alone. However, the interpretation is bolstered by the substantially greater prevalence of choice signals in CIP and the finding that choice signals moderated the strength of sensorimotor associations (which were stronger in CIP) (34–36). Finally, we found that the strength of within-domain clustering did not depend on whether the neurons showed mixed selectivity.

Considering that V3A receives direct input from V1 and V2 (25, 26) and is traditionally classified as a visual area, it may be surprising that it showed stronger clustering of saccade-related activity than CIP. It is therefore important to emphasize that extraretinal signals modulate visual responses in V3A, that saccade-related activity in V3A predicts the direction and timing of saccades, and that V3A shows sensorimotor associations between visual and saccade direction preferences (albeit weaker than in CIP) (36–41). The present data are consistent with a predominantly bottom-up saccade signal given that the activity began 6 ms earlier in V3A than in CIP and that the time course of CIP activity closely matched the temporally integrated V3A activity (36). However, other work suggests that corollary discharge supports saccadic remapping in V3A (41). These findings collectively suggest that V3A and CIP contain bottom-up and top-down saccade signals. Clarifying the origins of oculomotor signals in these areas and how they support visuomotor behavior will require further studies to characterize pre- and postsaccadic activity and whether that activity depends on the task or training history. Our findings further implicate V3A in oculomotor processing, support the reclassification of V3A as association cortex, and suggest that classical notions of sensorimotor dichotomies break down faster as information ascends the dorsal visual pathway than is generally thought.

The PPC of multiple species is widely implicated in transforming sensory information into decisions and motor responses (11, 56–60). In contrast to a linear cascade from sensory to motor representations across the cortical hierarchy, the present findings suggest that parallel processing of sensory and motor-related signals can occur within the same lower-level area, after which downstream, higher-level targets synthesize that information to bridge perception and action. In particular, the prevalence of mixed visual and saccade signals in V3A and CIP did not substantially differ, but the greater prevalence of choice signals in CIP was associated with stronger sensorimotor associations (35, 36). Among other PPC areas and functions, it is conceivable that similar architectures support evidence accumulation, categorical learning, and motor planning in the lateral intraparietal area (13, 61–63), the coordination of hand movements based on visual and proprioceptive signals in the medial intraparietal area (64, 65), as well as spatial and self-motion processing in the ventral intraparietal area (18, 66, 67). Testing this possibility will require the prevalence of mixed selectivity across hierarchical areas to be contrasted with the strength of sensory-sensory and/or sensory-motor associations.

Previous studies reported functional correlations between V3A/CIP activity and behavioral choices during 3-D discrimination tasks (31, 34–36). However, there is little evidence for causal relationships between these areas and 3-D perception. To our knowledge, no study has tested the perceptual effects of causally manipulating V3A. Two studies tested the perceptual effects of reversibly inactivating CIP with muscimol, but the results were limited. In the first study, the injections impaired performance in a delayed match-to-sample task for surface tilt but in only half of the experiments (68). In the second study, the injections resulted in a relatively small deficit in 3-D curvature discrimination (45). Because the perceptual effects of causal manipulations depend on clustering (69), the present findings provide essential information for studies assessing the causal roles of these areas in sensory and motor processing. In particular, the findings support the feasibility of such experiments, so long as the cross-area differences in clustering of visual selectivity, choice-related activity, and saccade-related activity are considered. For example, the differences may translate into larger 3-D perceptual effects when V3A is causally manipulated despite the fact that CIP is functionally more strongly associated with 3-D visual processing (27, 34–36). This may also relate to the limited behavioral effects of CIP inactivation in previous studies.

Some of our analyses showed that the similarity of a given property was comparable in the two areas, but clustering was statistically significant in only one. For instance, the summary statistics for the pose discrimination index in V3A and CIP were nearly identical, but clustering was only significant in V3A. Such cases highlight that clustering measures based on differences in property values must be interpreted relative to the underlying distribution of values. For example, random sampling of pairs from a narrower underlying distribution will produce smaller average pairwise differences than if the underlying distribution was broader. As such, the magnitude of pairwise differences alone is not sufficient to draw conclusions about clustering. Moreover, the dependence of such measures on the underlying distributions implies that they generally cannot be used to perform direct comparisons of clustering across areas. Instead, it is necessary to determine whether the observed differences are smaller than expected by chance given the underlying distribution (e.g., using resampling methods). For this reason, our cross-area comparisons were based on the correlations between the tuning curves of neuronal pairs and the proportion of properties with significant clustering.

The use of tetrode recordings facilitated the assessment of functional clustering based on direct comparisons of single-neuron responses. Although our focus was on the local similarity of tuning properties, our findings are consistent with the possibility of topographic maps of visual, choice, and saccade preferences. However, it was not possible to test for such maps because the penetration trajectories were always dorsal-ventral, resulting in approach vectors that were often not ideal for estimating layer/columnar information. The columnar organization of V3A and CIP can nevertheless be investigated with laminar probes like those used here by carefully selecting trajectories according to the convolutions of the cortical sheet. As such, the mesoscopic organization of visual, choice, and saccade properties in V3A and CIP as well as any relationships between feature maps remain topics for future studies.

## DATA AVAILABILITY

The data analyzed in this article are available through the Open Science Framework via our laboratory’s profile at https://osf.io/8wxk7/.

## GRANTS

This work was supported by NIH Grants EY029438 and EY035005. Z.Z. was partially supported by a Walsh Graduate Student Support Initiative award from the McPherson Eye Research Institute. B.K. was partially supported by NIH Grant NS128586. R.D. was partially supported by NIH Grant EY027721 and National Science Foundation (NSF) Grant DGE-1545481. A.R. was partially supported by NIH Grant NS105602. Further support was provided by NIH Grant P51OD011106 to the Wisconsin National Primate Research Center.

## DISCLOSURES

No conflicts of interest, financial or otherwise, are declared by the authors.

## AUTHOR CONTRIBUTIONS

B.K., R.D., T-Y.C., and A.R. conceived and designed research; B.K., R.D., and T-Y.C. performed experiments; Z.Z., B.K., R.D., and A.R. analyzed data; Z.Z., B.K., R.D., and A.R. interpreted results of experiments; Z.Z. and A.R. prepared figures; Z.Z. and A.R. drafted manuscript; Z.Z., B.K., R.D., T-Y.C., and A.R. edited and revised manuscript; Z.Z., B.K., R.D., T-Y.C., and A.R. approved final version of manuscript.
